# Medicinal and Aromatic Plants Used in Traditional Treatment of the Oral Pathology: The Ethnobotanical Survey in the Economic Capital Casablanca, Morocco (North Africa)

**DOI:** 10.1007/s13659-018-0194-6

**Published:** 2018-11-27

**Authors:** Sophia Zougagh, Ayoub Belghiti, Tarik Rochd, Ilham Zerdani, Jamal Mouslim

**Affiliations:** 1 Laboratory of Ecology and Environment, Faculty of Sciences Ben M’sik, University Hassan II Casablanca, Sidi Othman, BP 7955, Casablanca, Morocco; 2 Laboratory of Oral Biology, Faculty of Dentistry, University Hassan II Casablanca, Mers Sultan, BP 9157, Casablanca, Morocco

**Keywords:** Ethnobotanical survey, Medicinal and aromatic plants, Oral pathology, Traditional herbalists

## Abstract

**Abstract:**

In order to identify the medicinal and aromatic plants most requested for the treatment of the most common oral pathology, an ethnobotanical survey was carried out in the economic capital Casablanca, Morocco. The data basis was obtained draw selected traditional herbalists based on the semi-structured questionnaire. Quantitative indices such as use value (UV), family UV (FUV), fidelity level and informant consensus factor (ICF) were intended to evaluate the importance of plant species. A total of 46 plants species belonging to 22 families that were used. Juglandaceae family showed the highest significance (FUV = 0.75). We identified 40 species used for gum disease (gingivitis, periodontal abscess), 15 for dental pain (toothache, tooth sensitivity), 14 for halitosis, 12 for oral ulcers (aphtous, mouth ulcers and herpes), 3 for dental stain (teeth cleaning, sparkling and bleaching) and only 2 for tooth decay. The used plants are mainly prepared as decoction (80.4%). *Syzygium aromaticum* (UV = 0.94) was the specie most commonly prescribed by local herbalists. The higher ICF (0.75) was registered for the use gum disease.

**Graphical Abstract:**

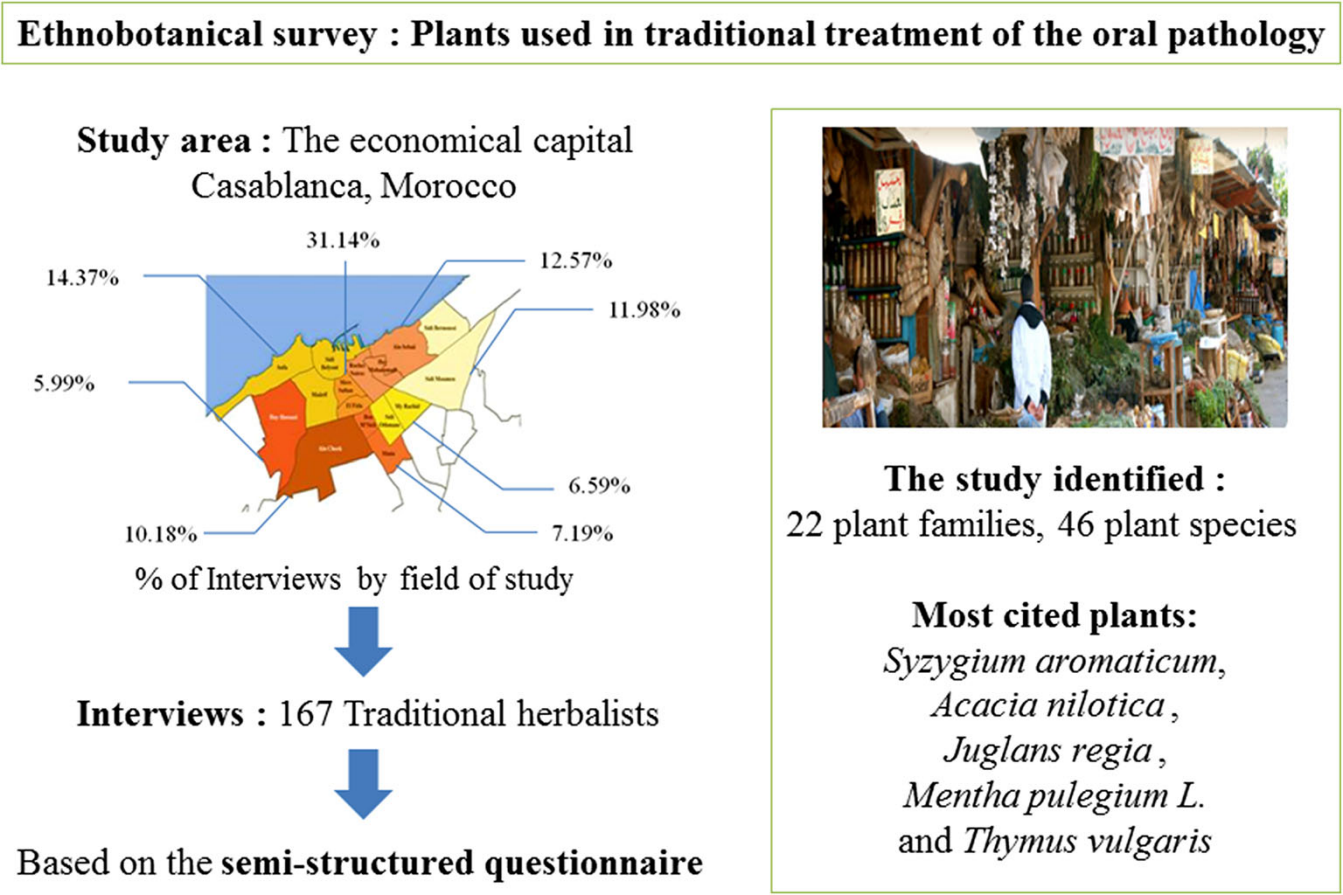

**Electronic supplementary material:**

The online version of this article (10.1007/s13659-018-0194-6) contains supplementary material, which is available to authorized users.

## Introduction

Oral health is an integral part of general health [1–3]. According to the World Health Organization (WHO), the burden of oral disease is a major health problem at the international scale in the 21st century [4, 5]. Additionally, the oral disease is still a major public health problem in high-income countries, as well; this problem is growing in many low- and middle-income countries [6]. To specify these problems, it found that the tooth decay, periodontal diseases, oral cancer, pharynx, and oral tissue lesions are important health problems [7].

In the world, nearly 100% of adults and 60 to 90% of schoolchildren, who have dental decay, often causing pain and discomfort. In addition, 20% to 50% of middle-aged adults (35–44 years) suffer from the severe periodontal disease, which can lead to tooth loss [6, 8]. In Morocco, the prevalence of caries is 86.9%, the DAO index (or average number of teeth Decayed, Absent, or Obturated) is 13.2% and periodontal diseases affect 83.9% of adult subjects. These results show a lack of knowledge of the rules of oral hygiene, an important need for preventive, conservative and prosthetic dental treatments [9].

Unlike infections in other parts of the body, oral infections are polymicrobial, which can exert at the same time their pathogen and making treatment difficult [10]. Furthermore it, they can have repercussions on the general state of the individual, favoring the appearance of certain attacks such as cardiopathy, gastropathy, and certain neuralgias.

These affections only benefit from a limited interest in Morocco. Oral health is often marginalized in developing countries, and attention is paid to severe and disabling diseases, making the traditional medicine popular to be very important in Morocco. Additionally, there is a family self-medication system in each geographical region, using traditional healers.

In conclusion, medicinal plants remain widely used in Morocco. In other words, a big part of traditional Moroccan medicine comes from Arab, Berber, Andalusian and African medicines adding the innovations brought by local populations [11]. Generally, in Africa, more than 80% of the population uses traditional medicine and medicinal plants for primary health care [12].

The plant properties used in traditional medicine are also considered as a subject of the field of dentistry to be exploited for the purpose of relieving tooth pain, gum inflammation, and canker sores or aphthous [13]. But firstly, it is more important to understand the effects of plant extracts on the human body and its interactions with other medicines. As well-known many of these extracts have anti-inflammatory effects and prevent bleeding, which can be the basic resources in the dental treatment [14]. We note that there are other effects of these plants such as antiseptics, antibacterial, antimicrobial, antifungal, antioxidant, antiviral, and analgesic agents [15].

The purpose of this work is interested in an inventory of plant species used in traditional medicine for the management of the most common diseases related to the oral cavity by the population of Casablanca. This will serve as a source of information on the use of plants in the field of dentistry, research data in the fields of phytochemistry and pharmacology and in the search for new natural molecules and combination for the treatment of oral pathology.

## Materials and Methods

### Study Area and Population

**Fig. 1:**
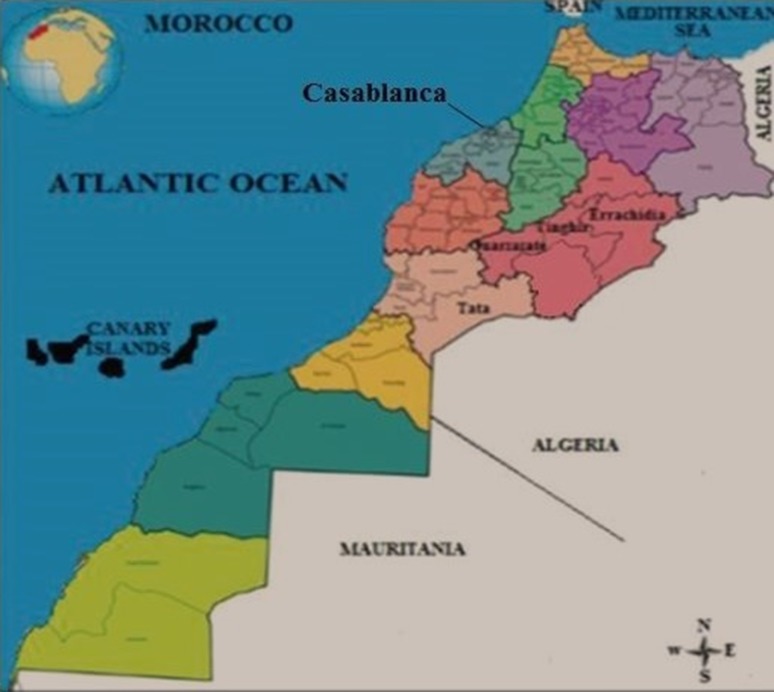
Localization of study area “Casablanca”

Casablanca is the economic city of Morocco, the capital of Casablanca-Settat region, is a financial capital and the first business center in Morocco. It is undoubtedly the largest city and it is located on the Atlantic coast, about 80 km at the south of Rabat, the administrative capital. Casablanca (33°32′00″N 7°35′00″W) is located on the plain of Chaouia-Ouardigha, one of the main centers of agricultural activity in the country [16] (Fig. 1). It is bounded by the Atlantic Ocean to the west and the Chaouia-Ourdigha region to the north, east and south (Province of Settat to the east and to the south and Ben Slimane province to the north). The climate of Grand Casablanca is oceanic: mild, rainy in the winter, humid, and temperate in the summer with no winter frost and high humidity during the year [17].

The Grand Casablanca is the most populated area of Morocco and hosts 12% of the total population of the country. It spans over 1115 km^2^ and counts 3615903 inhabitants, rather more women (1833648) than men (1782255); 63% of the population is under 35 years of age [18]. The population of the Greater Casablanca district is mainly urban (91.6% vs. 8.4% rural) and is concentrated in the city of Casablanca. The population of Morocco as a whole is less urban than the Greater Casablanca district (55% vs. 45% rural) [19].

However, Casablanca attracts a lot of migrants from all regions of Morocco, including the rural regions and these results in a very large socio-economic heterogeneity. The Grand Casablanca population can, therefore, be considered as reasonably representative of the Moroccan population. This may reflect diversity and richness in several domains, including the domain of traditional phytotherapy.

### Data Collection

Ethnobotanical data were collected from December 2016 to March 2017. Information about the medicinal use of plants was collected by carrying out semi-structured interviews with 167 traditional herbalists. Interviewees were selected by convenience sampling. The inclusion criteria were herbalists who prescribe plants for dental and oral problems. The exclusion criteria were herbalists who are limited only to the sale of medicinal plants and herbalists who do not prescribe medicinal plants for oral pathologies. We asked all herbalists located in their grocery stores.

The questionnaire was designed to collect data on:

(i) Socio-demographic information of the interviewees: Sex, age, length of experience, academic level and origin of oral health information.
(ii) Plants used in the treatment of oral pathologies: Concerns vernacular names, traditional uses, parts used, form of preparation, method of administration, posology, type of plant and price.
(iii) User Profile (Patients): Gives information on the age and sex of users who treat oral pathologies by plants prescribed by traditional herbalists.

The Arabic and Amazigh languages were used for collecting the data. The criteria used for selecting a medicinal and aromatic plant as a specific treatment was that this plant should be mentioned by more than five independent interviewees.

Data obtained during the survey were cross-checked vernacular names/scientific names according to published literature. Scientific names of species were identified following Greuter et al. Med-CheckList [20], Flora europaea 1 [21] and the plant list database [22] (Table 3). The nomenclature reference and the Med- ChekListe numero (MCL No) (Table S.1).

### Data Analysis

A descriptive statistical method using frequencies and percentages was used to analyze the socio-demographic data of the respondents (traditional herbalists), and the results of the ethnobotanical survey were analyzed using the Use Value (UV), Family UV (FUV), Fidelity Level (FL) and Informant Consensus Factor (ICF).

#### Use Value (UV)

The UV was used to determine the level of use of each species in the study area. It was calculated using the following formula:

$${\text{UV}} = \frac{{\text{Ui}}}{{\text{Ni}}}$$

where Ui = Number of use reports cited by each informant for a given species and Ni = Total number of informants. Use values are high when there are many use reports for a plant, implying that the plant is important, and approach zero (0) when there are few reports related to its use. The use value, however, does not distinguish whether a plant is used for single or multiple purposes [23–25].

#### Family UV (FUV)

The FUV identify the significance of plants families. It is as an index of cultural importance which can be applied in ethnobotany to calculate a value of biological plant taxon. To calculate FUV, we use the following formula:

$${\text{FUV}} = \frac{{\text{UVs}}}{{\text{Ns}}}$$

with UVs = UV of the species and Ns = Total number of species within each family [26].

#### Fidelity Level (FL)

FL was used to classify the recorded plant species based on their claimed relative effectiveness. We calculated FL using the following formula:

$${\text{FL}} = \frac{{\text{Np}}}{{\text{N}}} \times 100$$

where Np = Number of informants that claim a use of a plant species to treat a particular category and N = Number of informants that use the plants as a medicine to treat any given category [26, 27].

#### Informant Consensus Factor (ICF)

This measure was calculated for each category of disease to identify the level of agreement among the respondents on the reported medicinal plants used to cure a particular disease. The ICF was calculated using the following formula:

$${\text{ICF}} = \frac{{{\text{Nur}} - {\text{Nt}}}}{{{\text{Nur}} - 1}}$$

where Nur = Number of citations for each particular disease and Nt = Number of species reported to cure that disease [28]. This is an indication of agreement of informants for a plant species for a particular condition. Values are low (near 0) if plants are chosen randomly or if there is no exchange of information about their use among informants and approach one (1) when there is a well-defined selection criterion in the community and/or if information is exchanged between informants [29, 30].

## Results and Discussion

### Socio-demographic Information of the Interviewees

**Fig. 2:**
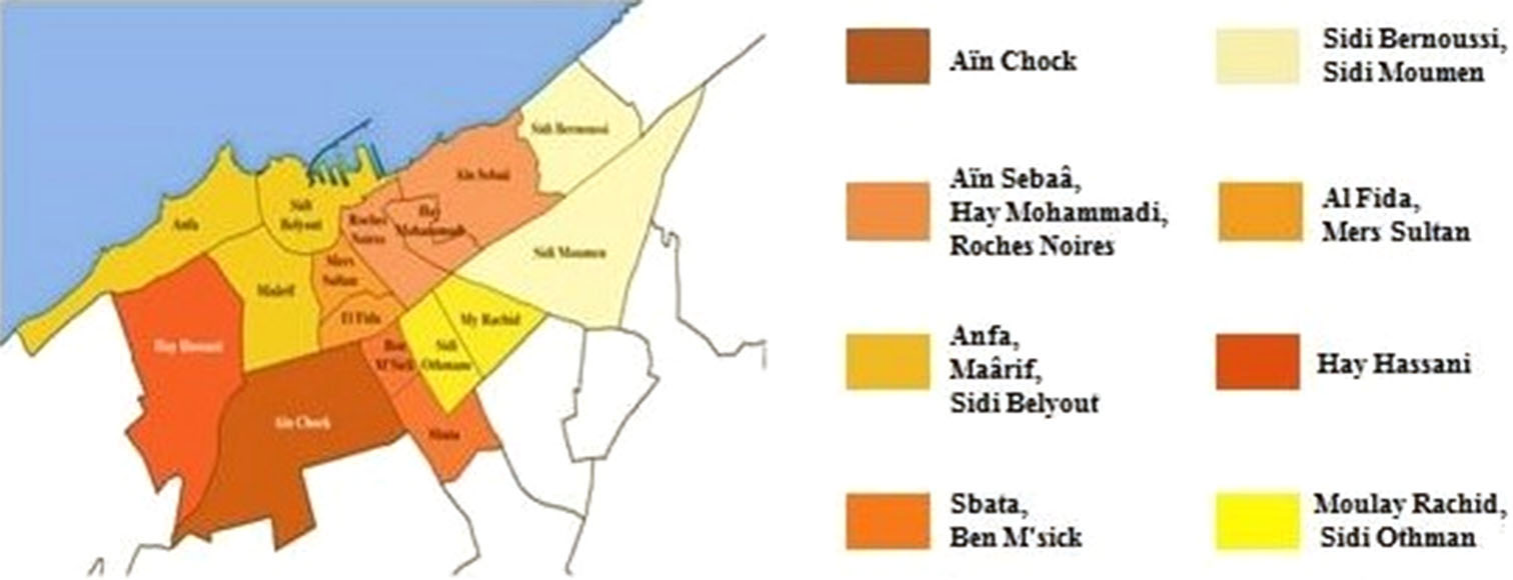
Map of the selected locations for the study

The number of respondents varies according to the area of study (Table 1; Fig. 2). Is composed of n = 167 traditional herbalists, which correspond to the main characteristics of the study population, in order to have a better representation of the results.

**Table 1:** Distribution of 167 traditional herbalists in Casablanca

Localities in the study area	Frequency	Percentage (%)
Aïn Chock	17	10.18
Aïn Sebaâ, Hay Mohammadi, Roches Noires	21	12.57
Anfa, Maârif, Sidi Belyout	24	14.37
Sbata, Ben M’sick	12	7.19
Sidi Bernoussi, Sidi Moumen	20	11.98
Al Fida, Mers Sultan	52	31.14
Hay Hassani	10	5.99
Moulay Rachid, Sidi Othmane	11	6.59

As shown in the Table 2, most of the respondents were male (92.22%) accounted of the study population, compared to 7.78% of female, with a sex ratio (male/female) of 11.85 (Table 2). This high percentage of men herbalists shows that the sale of medicinal plants remains predominantly a male domain in Morocco. Moreover, the documentation of traditional knowledge as part of plant biodiversity research has historically been gender-biased towards men, which can result in misleading and incomplete results [31–33]. Several studies have reported that focus on male specialists, has ignored a wealth of lay, female plant knowledge in ethnobotanical research [31, 33]. Although women are often more knowledgeable about medicinal plant identification and use than are men, and their knowledge can be epistemologically different [31]. Historically, ethnobotanists have been predominantly male, which hampered their access to women’s knowledge in societies where men are granted greater public access than women [33]. These considerations are particularly important in conducting ethnobotanical research in the world; women in urban contexts have so far gone unnoticed by ethnobotanical enquiry, although they are the main medicinal plant users [34].

**Table 2:** Sociodemographic characteristics of the traditional herbalists

Biodata	Frequency	Percentage (%)
Sex		
Female	13	7.78
Male	154	92.22
Age		
≤ 30	40	23.95
31–40	32	19.16
41–50	35	20.96
≥ 51	60	35.93
Educational attainment		
Informal	65	38.92
Primary	44	26.35
Secondary	40	23.95
University	18	10.78
Source of information		
Empirical	58	34.73
Hereditary	72	43.11
Formation	37	22.16

The age extremes of the herbalists ranged between 18 and 82 with an average age of 50 ± 10 years, the majority of them (35.93%) belonged to the age group over than 51 years (Table 2). Which shows that people of old age are the main custodians of traditional knowledge. This, however, poses a serious threat to the indigenous knowledge because it may eventually be lost following the demise of the older generation. This result is similar to the findings reported by Kankara et al. [35], This implies that if speedy interventions to incorporate them into oral health education and promotion efforts are not made, the legacy of the use of traditional medicines in the treatment of oral lesions may be lost [36]. Moreover the accumulated experience with age is the main source of information at the local level about the use of plants in traditional medicine.

The overage of years of experience in traditional therapeutics for herbalists was 10.58 ± 6,62 years (Table 2). Difference between age and length of experience was no significant (P = 0.88). The knowledge of the uses of medicinal plants and their properties is generally acquired after a long experience, accumulated and transmitted from one generation to another.

Regarding the level of education, 38.92% of the population was informal, the 50.3% of the remaining herbalists were divided between primary schooling (26.35%), secondary schooling (23.95%), and only 10.78% of the herbalists had graduate levels. The most senior traditional herbalists were illiterate, but form the majority of the trainers and are respected community leaders; therefore there is the existence of knowledge transfer from the elderly to younger healers and herbalists.

The majority of herbalists (77.84%) reported that they had never received any professional formation in their field. They acquired the information through the experiences of other herbalists and from their ancestor. This is information that is transmitted by heredity (Table 2). The consequence of this is that most knowledge on herbal remedies is handed down by older members of the community (over 51 years) it also proved that ethnomedicinal knowledge is concentrated among the senior members of the families, and it is relatively difficult to transfer the knowledge from the elderly to the younger generation [37]. There is also a loss of information on medicinal plants, which can be explained both by the reduction of plant resources in the region and by the mistrust of certain people, especially young people, who tend not to believe this traditional medicine [38]. At present, the traditional medical knowledge transmitted from generation to generation is in danger, because transmission between old people and younger generation is not always assured [39, 40].

### Plant Species Used for Traditional Treatment of Oral Pathologies

The Table 3 shows the plant species most popular prescribed by traditional herbalists for the treatments of oral diseases. The table shows the family and scientific names, vernacular name, the parts used, their form of preparation, the mode of administration, the traditional uses and the quantitative value CN, UV and FUV.

**Table 3:** List of Medicinal and aromatic plants used for the treatment of oral pathology prescribed by traditional herbalists

Family scientific Scientific name	Vernacular name	Part used	Mode of Preparation	Route of Administration	Traditional therapeutic effect	CN	UV	FUV
Amaryllidaceae								0.2
*Allium sativum* L.	Thouma	Clove	Cataplasm	Direct application	GD, DP	33	0.2	
Anacardiaceae								0.09
*Pistacia lentiscus* L.	Drou	Leaves/Flower	Decoction	Mouthwash	GD	15	0.09	
Apiaceae								0.1
*Ammi visnaga* (L.) Lam.	Bechnikha	Fruits	Raw/Decoction	Mouthwash/Direct application	GD, H, DP	28	0.16	
*Coriandrum sativum* L.	Kozbore	Leaves	Raw	Direct application	DP	11	0.06	
*Foeniculum vulgare* Mill.	El besbas	Leaves/Seeds	Decoction	Mouthwash	H	18	0.10	
*Pimpinella anisum* L.	Nafae	Seeds	Decoction/Infusion	Mouthwash	GD, H	12	0.07	
Apocynaceae								0.09
*Nerium oleander.*	Defla	Leaves/Root	Raw/Cataplasm	Mouthwash/Direct application	GD, DP, H	16	0.09	
Asteraceae								0.13
*Chamaemelum* *nobile*(L.) All.	Baboneje	Flower	Decoction	Mouthwash	GD, OrU, DP	45	0.27	
*Artemisia absinthium* L.	Chiba	Leaves	Decoction/Infusion	Mouthwash	GD, DP	17	0.10	
*Artemisia herba alba* Asso./*A. vulgaris* L./*A. mesatlantica* Maire in Bull.	Chih	Leaves/Flower bud	Decoction	Mouthwash	GD	20	0.12	
*Atractylis gummifera.*	Addad	Root	Raw	Direct application	DP	11	0.06	
Cupressaceae								0.07
*Juniperus phoenicea* L.	Arar	Leaves	Decoction	Mouthwash	GD	18	0.10	
*Thuja occidentalis* L.	Afsa	Leaves	Decoction	Mouthwash	GD	9	0.04	
Fabaceae								0.71
*Acacia nilotica.*	Sallaha	Clove	Decoction/Raw	Mouthwash/Direct application	GD, H, DS	155	0.92	
*Glycyrrhiza glabra* L.	Arksous	Rhizome	Raw	Brushing	GD, DS	85	0.5	
Fagaceae								0.13
*Quercus suber* L.	Dbagh	Root	Decoction	Mouthwash	GD	23	0.13	
Iridaceae								0.34
*Crocus sativus* L.	Zaafran hor	Stigma	Decoction	Mouthwash	GD	58	0.34	
Juglandaceae								0.75
*Juglans regia* L.	Souak	Bark of the root	Raw/Cataplasm	Brushing/Gum	GD, H, DS	126	0.75	
Lamiaceae								0.33
*Calamintha officinalis*	Manta	Leaves	Decoction	Mouthwash	GD, H	40	0.23	
Moench								
*Lavandula vera.*	Khzama	Leaves/Flower	Decoction	Mouthwash	GD	56	0.33	
*Marrubium vulgare* L.	Meriouth	Leaves/Whole	Decoction	Mouthwash	GD, DP	34	0.2	
*Melissa officinalis* L.	Naanaa soufi	Leaves	Decoction	Mouthwash	GD, OrU	95	0.56	
*Mentha pulegium* L.	Flio	Leaves	Infusion/Essential oil	Mouthwash/Direct application	GD, H, DP, TD	123	0.73	
*Mentha suaveolens* Ehrh.	Timija	Leaves	Decoction	Mouthwash	GD	24	0.14	
*Ocimum basilicum* L.	Hbak	Leaves	Decoction	Mouthwash	OrU	14	0.08	
*Origanum majorana* L.	Merdedouche	Leaves/Whole	Decoction	Mouthwash	GD, DP	38	0.22	
*Origanum vulgare* L.	Zaater	Leaves	Decoction	Mouthwash	GD, OrU, H	46	0.27	
*Rosmarinus officinalis* L.	Azir	Leaves	Decoction	Mouthwash	GD	56	0.33	
*Salvia officinalis* L.	Salmia	Leaves	Decoction	Mouthwash	GD, OrU	83	0.5	
*Teucrium polium* L.	Jaiidia	Leaves	Decoction	Mouthwash	GD	17	0.1	
*Thymus vulgaris* L.	Ziitra	Leaves	Decoction/Essential oil	Mouthwash/Direct application	GD, OrU, H	108	0.64	
Lauraceae								0.44
*Cinnamomum zeylanicum.*	Karfa	Bark of the trunk	Decoction/Essential oil	Mouthwash/Direct application	GD, OrU	81	0.48	
*Laurus nobilis.*	Wrap sidna moussa	Leaves	Decoction/Infusion	Mouthwash	GD, H	69	0.41	
Lythraceae								0.28
*Lawsonia inermis.*	Henna	Leaves	Raw	Direct application	OrU	7	0.04	
*Punica granatum* L.	Romane	Bark of the fruit/Flower	Decoction	Mouthwash	GD	88	0.52	
Myrtaceae								0.55
*Eucalyptus globulus* Labill.	Kalitouse	Leaves	Decoction/Infusion	Mouthwash/Direct application	GD, OrU, DP	73	0.43	
*Myrtus communis* L	Rihane	Leaves	Decoction	Mouthwash	GD, DP, H	51	0.3	
*Syzygium aromaticum.* (L.) Merr. & L.M.Perry	Krounfel	Flower bud	Raw/Essential oil	Mouthwash/Direct application/Gum	GD, DP, H, TD	158	0.94	
Nitrariaceae								0.10
*Peganum harmala* L.	Harmal	Seeds	Decoction	Mouthwash	GD, H, DP	18	0.10	
Oléaceae								0.35
*Olea europaea* L.	Zitoune	Leaves	Decoction/Infusion/Raw	Mouthwash/Gum	GD, OrU, DP	59	0.35	
Pteridaceae								0.14
*Adiantum capillus*-*veneris* L.	Ziata	Leaves	Decoction	Mouthwash	GD	24	0.14	
Ranonculaceae								0.09
*Nigella sativa* L.	Sanouj	Seeds	Decoction	Mouthwash	GD, DP	16	0.09	
Rosaceae								0.03
*Eriobotrya japonica* (Thunb.) Lindl.	Mzah	Leaves/Bark of the stem	Decoction	Direct application	OrU	6	0.03	
Rutaceae								0.04
*Ruta montana* (L.) L	Fijel	Leaves	Decoction	Mouthwash	GD	7	0.04	
Salicaceae								0.2
*Populus nigra* L.	Safsaf	Leaves	Decoction	Mouthwash	GD	34	0.2	
Urticaceae								0.09
*Urtica urens* L.	Horiga elmelsa	Leaves	Decoction	Mouthwash	GD, OrU	16	0.09	

*GD* gum disease, *DP* dental pain, *H* Halitosis, *OrU* oral ulcers, *DS* dental stain, *TD* tooth decay, *CN* number of informant who cited a given plant species, *UV* use value, *FUV* family use value

**Fig. 3:**
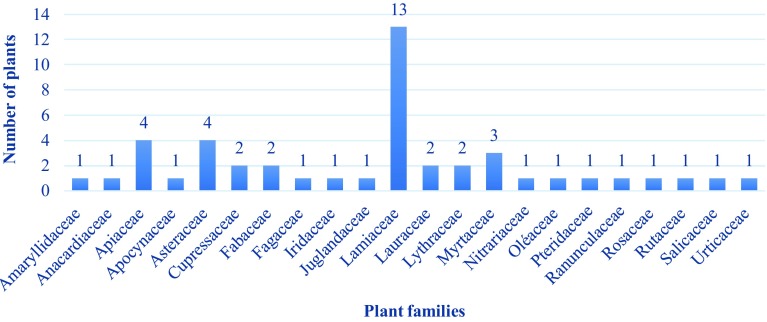
Distribution of plants species according to botanical families

The collected data identified forty-six (46) plant species belonging to twenty-two (22) botanical families (Fig. 3), the most represented being Lamiaceae, Apiaceae, Asteracea and Myrtaceae: the medicinal flora is dominated mainly by Lamiaceae and Asteraceae, which are among the nine main families of the spontaneous flora of Morocco, with a large number of species Mediterranean studies show that Asteraceae and Lamiaceae are used in traditional medicine [38, 41, 42]. They are also the predominant family of traditional medicine in West Bengal in India despite the tropical climate [43]. Most of the families recorded are represented by many species which shows that medicinal plants used are not concentrated only in a few families and genera. This agrees with other ethnobotanical studies carried out in Morocco and in Mediterranean area [44–48]. Many properties are, indeed, attributed to Lamiaceae in particular, anti-inflammatory properties, antiviral, antibacterial, antiallergic and antioxidant [49–51]. These different properties are due to their chemical constituents interesting from the point of view pharmacological. These are tannins, coumarines, mucilages, flavonoïds and phenolic acids suchas rosmarinic acid [52]. Furthermore, this family is characterized by the presence of oils essential elements that have found a great place in therapeutics thanks to their broad spectrum of biological activities [53], [54].

The vernacular name of a plant could correspond to several species at once, although of the same kind. This could be the source of the inefficiency of the treatments in particular that the chemical composition already varies with the edaphic or climatic races of the same species, as well qualitatively as quantitatively and thus it would surely be amplified between the species of the same kind. Note that the same species can treat several pathologies. And it’s necessary to inform and heighten public awareness about toxic plants in order to keep off, at least, some accidental intoxication due to ignorance of plants. In the same way, the passing on of medical knowledge is done according to oral initiatory chain, which led to some impoverishment of doctrinaire knowledge.

This ethnobotanical analysis revealed that the majority of plants recipe are used for gum disease (gingivitis, periodontal abscess) with a total of 40 plant species, followed 15 for dental pain (toothache, tooth sensitivity), 14 for halitosis, 12 for oral ulcers (aphtous, mouth ulcers and herpes), 3 for dental stain (teeth cleaning, sparkling and bleaching) and only 2 for tooth decay (Table 3). Lewis et al. [55] reported on the oral health care knowledge and practices of African traditional herbalists from two communities (Zonkizizwe and Dube in the Gauteng Province, South Africa). According to their findings, more than 90% of traditional herbalists from both areas correctly identified photographs of gingival inflammation, dental caries and oral candidiasis. More than half reported patients presented with mouth problems such as toothache, swollen gums and oral candidiasis.

Different parts of the plants are used; leaves are the most used parts with 67.4% followed by bark from different part (trunk, root, fruit and stem) with in total 8.8%, flowers and seeds (8.7%), roots (6.5%), clove, flower bud and whole plant (4.3%) and fruits, rhizome, stigma with 2.2% (Table 3). The leaves are the most exploited organs this can be explained by the fact that they are at the same time centers of photochemical reactions and reservoirs of organic matter derived from them; they provide the majority of alkaloids, glycosides and essential oils [56, 57]. The use of leaves can also be explained by the ease and speed of their harvest [58].

In order to facilitate the administration of the active ingredients of the plant, several form of preparation are used, namely the decoction is the most frequent form used with 80.4% followed by raw form (21.7%), infusion (13%), essential oil (8.7%) and cataplasm with 6.5% (Table 3). The form of decoction remains the most effective way that allows the extraction and assimilation of active ingredients while disinfecting the plant; however, it may destroy some active ingredients [57]. The mode of preparation of a phyto-therapeutic product may have an effect on the amount of active ingredient present.

For treating some oral pathology, the plants were used alone or as a combination of two or more varieties in the same recipe. But remedies prepared from a single plant are preferred for their simplicity of preparation and to avoid toxicity.

Plants preparations used for the treatment of oral diseases in the traditional way are often used in the form of a mouthwash with 84.8%, direct application (28.3%) and by brushing or gum form with 4.3% for each method of administration (Table 3). Mouthwash is an easy to use form and allows access to areas that are difficult to reach. It is practiced after brushing for the hygiene of the teeth, the prevention of the pathologies, the gingivitis therapy, the esthetics and the well-being against the halitosis [59]. The ability of herbal extract, in mouthwashes to reduce gingival inflammation and plaque formation and to be used as an irrigation agent to disinfect the root canal with less toxicity, has been well documented [60, 61]. The roots of some plants are used in the form of a toothbrush stick.

The UV of the cited plante ranged between 0.04 and 0.94. The highest UV was recorded for the *Syzygium aromaticum* (0.94), *Acacia nilotica* (0.92), *Juglans regia* (0.75), *Mentha pulegium.* (0.73) and *Thymus vulgaris* (0.64). The FUV ranged between lowest value (0.04) and highest value (0.75). The Juglandaceae family (0.75) was the highest FUV recorded followed successively by Fabaceae with (0.71), Myrtaceae with (0.55), Lauraceae (0.44) and Lamiaceae (0.33) (Table 3).

The high values of UV and FUV can be explained by the fact that these plants are the best known and have long been used by the majority of informants, representing a source of reliability. In fact, many biological activity and phytochemical evaluation were carried out for these plants and these species are particularly interesting for research of bioactive compounds. The scientific basis of traditional uses of the most frequently used plants through literature research: *Syzygium aromaticum,* it had been shown that this plant possesses a potent antimicrobial and antibiofilm property due to the presence of eugenol [62]. *S. aromaticum* is used for the treatment of gingivitis [13], stomatitis [63] and dental pain [64]. *Acacia nilotica*, has been used as an oral hygiene adjuvant from ages, but still, studies are lacking on the antifungal property of this plant on the most common oral fungus. Pai et al. [65] show in an antifungal activity against *Candida* *albicans*. This plant contains an additional product known as methyl gallate, which possesses antimicrobial activity by accelerating DNA damage by a ferric-bleomycin system [66]. *Juglans regia* contains chemicals like ascorbic acid, juglone, folic acid, gallic acid, regiolone that are involved in the antibacterial, antioxidant, and antifungal activities of this plant. It has greater potential for treating more severe cases such as periodontitis and gingivitis due to its antibacterial effect on different microbial flora particularly, gram positive organisms and oral pathologic bacteria [67, 68]. *Mentha pulegium,* Effective substances of this plant include pulegone, menthone, isomenthone, piperitonone, limonene, menthol, hesperidin, diosmine and azolen [69]. This plant is used mainly for halitosis [70–72]. *Thymus vulgaris,* this plant is widely studied for its antibacterial effect in many oral diseases like gingivitis, stomatitis and halitosis [72] and more recently Khan et al. [73] reported its effect on cariogenic bacteria because of the presence of carvacrol and thymol.

Our study revealed four species against gum disease with high FL: *T. vulgaris L* (100%), *S. aromaticum* (97.05%), *M. pulegium* (85.26%) and *C. sativus* (63.72%), respectively. *C.* *nobile* and *S. aromaticum* note a FL of 100% and 98.03% respectively against dental pain. Two species against halitosis with highest FL (100%) were *M. pulegium* and * Syzygium aromaticum.* For oral ulcers two species *S. officinalis* and *C. zeylanicum* with FL of 63.72% and 56.86% respectively. Two species against dental stain with highest FL 100% were *J. regia * and *A. nilotica*. *S. aromaticum* against tooth decay with highest FL (100%) (Table 4). The high FL of a species indicates the prevalence of a specific disease in an area and the utilization of plant species by the inhabitants to treat it [74, 75].

**Table 4:** Most frequently used plants for different categories based on highest FL (%) and ICF values of category of disease

Pathology	Plants species	FL (%)	Nt	Nur	ICF
Dental pain			15	36	0.6
	*Chamaemelum* *nobile* (L.) All.	100			
	*Syzygium aromaticum* (L.).	98.03			
	*Origanum majorana* L.	76.47			
	*Eucalyptus globulus* Labill.	68.62			
Halitosis			14	31	0.56
	*Syzygium aromaticum* (L.).	100			
	*Mentha pulegium* L.	100			
	*Thymus vulgaris* L.	77.45			
	*Juglans regia* L.	58.82			
Gum disease			40	158	0.75
	*Thymus vulgaris* L.	100			
	*Syzygium aromaticum* (L.).	97.05			
	*Mentha pulegium* L.	85.26			
	*Crocus sativus* L.	63.72			
Oral ulcers			12	27	0.57
	*Salvia officinalis* L.	63.72			
	*Cinnamomum zeylanicum.*	56.86			
Dental stain			3	7	0.66
	*Juglans regia* L.	100			
	*Acacia nilotica.*	100			
Tooth decay			2	3	0.5
	*Syzygium aromaticum* (L.).	100			
	*Mentha pulegium* L.	44.11			

The ICF values obtained for the categorized uses are presented in Table 4. Six categories were reported, namely, gum disease, dental pain, halitosis, oral ulcers, dental stain and tooth decay. ICF values obtained for the reported categories indicate the degree of shared knowledge for the uses of medicinal herbs. The ICF’s factors ranging from 0.5 to 0.75 per uses categories. The ICF (0.75) was registered for the use gum disease category with 40 species, which may indicate a high incidence of this type in this region. The medicinal plants that are widely used by the local people have higher FL values than those that are less popular. On the other hand, medicinal plants that are known as remedies of a single aliment have 100% fidelity level than those that are used as remedies for more than one type of aliment.

The duration of the treatment varies according to the type of pathology thus for the gum disease the treatment varies between 1 and 3 weeks 2 to 3 times per day while for the oral ulcers, the halitosis and the dental stain the preparation of the plants is used until disappearance of effects. For dental pain treatment is used in case. The doses of the plants used are not precise generally are expressed by pinch, spoonful or handle. The dose is still random which is manifested by adverse effects on health because it says “no substance is poison itself; it is the dose that makes the poison”. Then, the patients did not take into account the accumulation of some constituents in body after a prolonged use of plants, which could provoke severe side effects and could also aggravate the disorder [76].

**Fig. 4:**
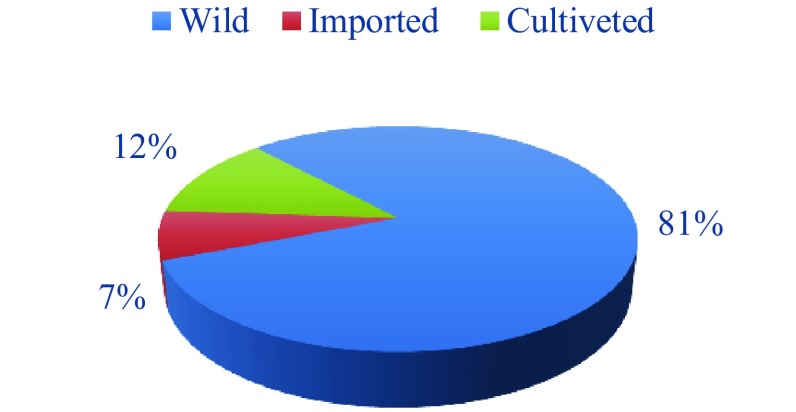
Domestication status of medicinal and aromatic plants used for traditional oral treatment

The type of plants used is 81% of the wild plants, cultivated plants with 12% and the imported plants represent only 7% (Fig. 4). In Morocco, the traditional pharmacopoeia disposes of a wide arsenal of plant remedies, because of the diversity of its environment and flora. It should be noted that the composition of a plant can vary from one specimen to another, depending on the terrain, growth conditions, humidity, temperature and sunshine. For the imported plants, the collection and the methods of conservation, stockage etc. can also alter the properties of the plants. Moreover dry plants sold in transparent bags should be avoided because the light partly affects their properties [77].

**Fig. 5:**
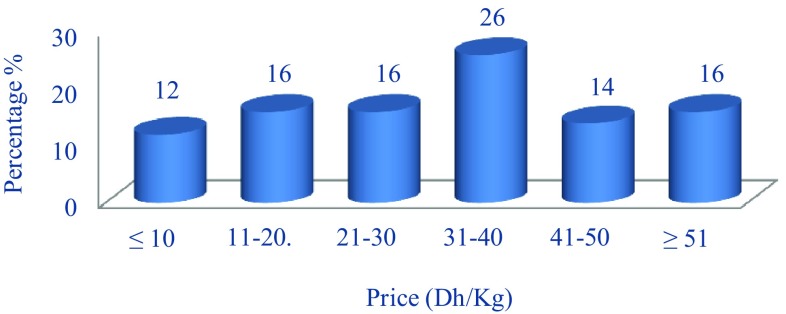
Prices of the most used medicinal and aromatic plants for traditional oral treatment

The prices of the most used plants vary between 31 and 40 Dh/kg with 26% followed by plants between 11–20 and 21–30 Dh/kg and more than 51 Dh/kg (16% for each category). Plants less than 10 Dh/kg represent only 12% (Fig. 5). In some countries, oral diseases are the fourth most expensive diseases to treat [78]. Few published data are available on the financial costs of traditional medicine in low- and middle-income countries. Although social, medical, and cultural reasons may account for why people in a given country prefer traditional medicine to conventional medicine, economic forces are also at play. The cost of treatment using medicinal plants is much cheaper than the cost of accessing a conventional medical service. The traditional use of medicinal plants is therefore the basis of curative medicine for low-income populations [79–81]. For example: *Syzygium aromaticum* gel can provide dentists with an alternative to benzocaine for topical anesthesia in their daily practice, especially for use with children and in areas where cost and availability limit access to pharmaceutical topical anesthetics [82].

## Conclusion

Our study highlighted the importance of the role of traditional herbal medicine in urban areas of Casablanca; we showed the importance of traditional medicine in the oral health system for the investigated populations. Data collected may help to preserve knowledge about different plants used and their mode of preparation. The results of this study have been obtained from the traditional herbalists in one of the most peopled region in North Africa, revealing the importance of the practice of plant medicine to treat oral diseases mainly as gum disease. They show that the traditional herbal medicine continues to be in demand from this region. This population confidence can be correlated to the efficiency of this source and/or its ability to be an alternative choice regarding the cost of modern medicine. In Morocco, the traditional medicines are still used and constitute in fact a very rich heritage, which is obligatory to keep. Phytotherapy should not be a poor medicine, but a real tool of medicines for all people. Therefore, we should study these drugs, in order to select the real therapeutic means. We suggest and recommend that documented plants to be screened for further ethnopharmacological studies. In order to translate this traditional knowledge, oral, into a scientific knowledge, leading to provide the invaluable compounds to be a starting point for the development of new drugs and wellness products.

## Electronic supplementary material

Below is the link to the electronic supplementary material.

Supplementary material 1 (DOCX 18 kb)

## Abbreviation

CN: Number of informant who cited a given plant species
FUV: Family use value
FL: Fidelity level
ICF: Informant consensus factor
UV: Use value
WHO: World Health Organization
