# Supplementary material for: Medicinal and Aromatic Plants Used in Traditional Treatment of the Oral Pathology: The Ethnobotanical Survey in the Economic Capital Casablanca, Morocco (North Africa)
Source: Nat Prod Bioprospect. 2018 Nov 27;9(1):35–48. doi: 10.1007/s13659-018-0194-6 (PMC6328421; doi:10.1007/s13659-018-0194-6)
Supplement: Supplementary file 1 — Supplementary material 1 (DOCX 18 kb) [file 13659_2018_194_MOESM1_ESM.docx]

**Medicinal and aromatic plants used in traditional treatment of the oral pathology: The ethnobotanical survey in the economic capital Casablanca, Morocco (North Africa).**

Sophia Zougagh ^a*^, Ayoub Belghiti ^a^, Tarik Rochd ^b^, Ilham Zerdani ^a^, Jamal Mouslim ^a^

*^a^ Laboratory of Ecology and Environment, Faculty of Sciences Ben M’sik, University Hassan II Casablanca, BP 7955 Sidi Othman, Morocco*

*^b^ Laboratory of Oral Biology, Faculty of Dentistry, University Hassan II Casablanca, BP 9157 Mers Sultan, Morocco*

**Supplementary Information**

**Table S.1.**

The nomenclature reference and the Med- ChekListe numero (MCL No)

| Famely  *Name.* | Nomencl. ref.  MCL. No. |
| --- | --- |
| Amaryllidaceae |  |
| *Allium sativum* L. | Sp. pl. 1:296. 1753 |
| Anacardiaceae |  |
| *Pistacia lentiscus* L. | Sp. pl. 2:1026. 1753  [129-46-526](http://ww2.bgbm.org/mcl/PTaxonDetail.asp?NameId=1195&PTRefFK=1273) |
| Apiaceae |  |
| *Ammi visnaga* (L.) Lam. | Sp. pl. 242. 1753 |
| *Coriandrum sativum* L. | Sp. pl. 256. 1753 |
| *Foeniculum vulgare* Mill. | Gard. Dict., ed. 8, n. 1. 1768 |
| *Pimpinella anisum* L. | Sp. pl. 263. 1753 |
| Apocynaceae |  |
| *Nerium oleander.* | Sp. pl. 209. 1753  [146-54-652](http://ww2.bgbm.org/mcl/PTaxonDetail.asp?NameId=1227&PTRefFK=1273) |
| Asteraceae |  |
| *Chamaemelum nobile* (L.) All. | Sp. pl. 894. 1753 |
| *Artemisia absinthium* L. | Sp. pl. 848. 1753 |
| *Artemisia herba alba* Asso*./* | Syn. Stirp. Arag.: 117.1779 |
| *A. vulgaris* L.*/* | Sp. pl. 2:848. 1753 |
| *A. mesatlantica* Maire in Bull | Soc. Sci. Nat. Maroc 8: 138. 1928 |
| *Atractylis gummifera.* | Sp. pl. 829. 1753 |
| Famely  *Name.* | **Nomencl. ref.**  **MCL. No.** |
| Cupressaceae |  |
| *Juniperus phoenicea* L. | Sp. pl. 1040. 1753 |
| *Thuja occidentalis* L. | Sp. pl. 2:1002. 1753 |
| Fabaceae |  |
| *Acacia nilotica.* | Sp. pl. 4: 1085. 1806  [490-04-638](http://ww2.bgbm.org/mcl/PTaxonDetail.asp?NameId=9116&PTRefFK=1276) |
| *Glycyrrhiza glabra* L*.* | Sp. pl. 741. 1753  [495-62-398](http://ww2.bgbm.org/mcl/PTaxonDetail.asp?NameId=11785&PTRefFK=1276) |
| Fagaceae |  |
| *Quercus suber* L. | Sp. pl. 995. 1753  [402-78-894](http://ww2.bgbm.org/mcl/PTaxonDetail.asp?NameId=27603&PTRefFK=1275) |
| Iridaceae |  |
| *Crocus sativus* L*.* | Sp. pl. 36. 1753 |
| [Juglandaceae](https://en.wikipedia.org/wiki/Juglandaceae) |  |
| *Juglans regia* L. | Sp. pl. 997. 1753 |
| Lamiaceae |  |
| *Calamintha officinalis* Moench. | Methodus 409. 1794 |
| *Lavandula vera.* | Sp. pl. 572. 1753 |
| *Mentha pulegium* L*.* | Sp. pl. 577. 1753  [477-36-780](http://ww2.bgbm.org/mcl/PTaxonDetail.asp?NameId=29414&PTRefFK=1275) |
| *Origanum majorana* L*.* | Sp. pl. 590. 1753  [477-64-568](http://ww2.bgbm.org/mcl/PTaxonDetail.asp?NameId=29705&PTRefFK=1275) |
| *Origanum vulgare* L*.* | Sp. pl. 590. 1753  [477-64-978](http://ww2.bgbm.org/mcl/PTaxonDetail.asp?NameId=29750&PTRefFK=1275) |
| *Rosmarinus officinalis* L*.* | Sp. pl. 23. 1753  [478-22-650](http://ww2.bgbm.org/mcl/PTaxonDetail.asp?NameId=29909&PTRefFK=1275) |
| *Salvia officinalis* L*.* | Sp. pl. 23. 1753  [478-26-652](http://ww2.bgbm.org/mcl/PTaxonDetail.asp?NameId=30062&PTRefFK=1275) |
| *Teucrium polium* L*.* | Sp. pl. 566. 1753  [478-66-748-74](http://ww2.bgbm.org/mcl/PTaxonDetail.asp?NameId=31654&PTRefFK=1275) |
| *Thymus vulgaris* L*.* | Sp. pl. 591. 1753  [478-72-978](http://ww2.bgbm.org/mcl/PTaxonDetail.asp?NameId=32131&PTRefFK=1275) |
| Lauraceae |  |
| *Cinnamomum zeylanicum.* | Bijdr. Fl. Ned. Ind. 11: 568. 1826 |
| *Laurus nobilis.* | Sp. pl. 369. 1753  [486-52-640](http://ww2.bgbm.org/mcl/PTaxonDetail.asp?NameId=9090&PTRefFK=1276) |
| Lythraceae |  |
| *Lawsonia inermis.* | Sp. pl. 349. 1753 |
| *Punica granatum* L. | Sp. pl. 472. 1753  [642-78-410](http://ww2.bgbm.org/mcl/PTaxonDetail.asp?NameId=19152&PTRefFK=1276) |
| Myrtaceae |  |
| *Eucalyptus globulus* Labill*.* | Voy. rech. Pérouse 1:153, t. 13. 1800 |
| *Myrtus communis* L. | Sp. pl. 471. 1753  [567-24-232](http://ww2.bgbm.org/mcl/PTaxonDetail.asp?NameId=15426&PTRefFK=1276) |
| *Syzygium aromaticum*(L.) Merr. & L.M.Perry | Mem. Amer. Acad. Arts ser. 2, 18:196. 1939 |
| [Nitrariaceae](https://www.google.com/search?q=Nitrariaceae&stick=H4sIAAAAAAAAAONgVuLSz9U3MMmtLDA1BwBi68F0DgAAAA&sa=X&ved=2ahUKEwiQzYbzia7eAhVS1hoKHRkmAoMQmxMoATAZegQIBBAH) |  |
| *Peganum harmala* L*.* | Sp. pl. 444. 1753 |
| Famely  *Name.* | **Nomencl. ref.**  **MCL. No**. |
| Oléaceae |  |
| *Olea europaea* L*.* | Sp. pl. 8. 1753  [581-66-364](http://ww2.bgbm.org/mcl/PTaxonDetail.asp?NameId=15545&PTRefFK=1276) |
| Pteridaceae |  |
| *Adiantum capillus-veneris* L. | Sp. pl. 2:1096. 1753  [051-14-164](http://ww2.bgbm.org/mcl/PTaxonDetail.asp?NameId=172&PTRefFK=1273) |
| Ranunculaceae |  |
| *Nigella sativa* L. | Sp. pl. 531. 1753  [646-64-824](http://ww2.bgbm.org/mcl/PTaxonDetail.asp?NameId=19929&PTRefFK=1276) |
| Rosaceae |  |
| *Eriobotrya japonica* (Thunb.) Lindl. | Trans. Linn. Soc. London 13:102. 1821 |
| Rutaceae |  |
| *Ruta montana* (L.) L. | Amoen. Acad. 3: 52, 1756 |
| Salicaceae |  |
| *Populus nigra* L. | Sp. pl. 1034. 1753 |
| Urticaceae |  |
| *Urtica urens* L. | Sp. pl. 985. 1753 |

MCL No: Med-CheckListe numero, Sp. pl: specimen. planche
